# The experience of premature birth for fathers: the application of the Clinical Interview for Parents of High-Risk Infants (CLIP) to an Italian sample

**DOI:** 10.3389/fpsyg.2015.01444

**Published:** 2015-09-29

**Authors:** Carla Candelori, Carmen Trumello, Alessandra Babore, Miri Keren, Roberta Romanelli

**Affiliations:** ^1^Laboratory of Dynamic Psychology, Department of Psychological Sciences, Health and Territory, Università degli Studi “G. d’Annunzio” Chieti-PescaraChieti, Italy; ^2^Infant Mental Health Unit, Geha Mental Health Center, Sackler Faculty of Medicine, Tel Aviv UniversityTel Aviv, Israel; ^3^Laboratory of Psychometrics, Department of Psychological Sciences, Health and Territory, Università degli Studi “G. d’Annunzio” Chieti-PescaraChieti, Italy

**Keywords:** anxiety, depressive symptoms, fathers, neonatal intensive care unit, prematurity

## Abstract

**Aim**: The study explored fathers’ experience of premature birth during the hospitalization of their infants, analyzing levels of depressive and anxiety symptoms as compared with mothers. Moreover the Italian version of the Clinical Interview for Parents of High-Risk Infant (CLIP) was tested through confirmatory factor analysis.

**Methods:** Couples of parents (*N* = 64) of preterm infants (gestational age < 37 weeks) were administered a socio-demographic questionnaire, the Edinburgh Postnatal Depression Scale, the State-Trait Anxiety Inventory and the CLIP after the admission to the Neonatal Intensive Care Unit (NICU).

**Results:** Significant levels of anxiety and depressive symptoms and high percentages of subjects above the corresponding risk thresholds were found among fathers and mothers with higher scores among the latters. Confirmatory factor analysis of the CLIP showed an adequate structure, with better fit for mothers than for fathers.

**Conclusion:** Results highlighted the importance for nurses and clinicians working in the NICU to consider not only the maternal difficulties but also the paternal ones, even if these are often more hidden and silent. In addition the CLIP may be considered an useful interview for research and clinical purposes to be used with parents of high-risk infants.

## Introduction

The birth of a child and the transition to parenthood represent a deep emotional experience for most parents and may cause stress in their life (Schappin et al., 2013). However, empirical research on parents of preterm infants has demonstrated that the premature birth and the subsequent admission to NICU are stressful experiences significantly different from that of parents of full term infants (Goldberg and DiVitto, 2002): the lack of preparation for parenthood, the hospitalization itself, the grief and the isolation all contribute to a very difficult emotional condition for mothers and fathers (Whittingham et al., 2014).

Preterm birth can be considered a traumatic event that involves child and parents (Tracey, 2000). Several studies on premature infants and their parents focused primarily on mothers, but recent research, that included fathers, suggest that both parents are emotionally involved (Matricardi et al., 2013; Tooten et al., 2013) and equally important in influencing infants’ social, behavioral, and psychological outcomes (Goldberg and DiVitto, 2002; Sarkadi et al., 2008; Ramchandani et al., 2013).

Considering the point of view of the father he may feel that he has to deal with the emotional aspects connected to his paternal role as well as with the need to play a supportive role in relation to his partner.

Many of the recent studies about the parents of prematurely born children show that a common worry of the fathers is about the “effort” required in playing their different roles: being the father of child, supporting the mother and providing for the needs of the family in such a difficult and painful situation (Pohlman, 2005; Lindberg et al., 2007).

For fathers, the preterm delivery and the visits to the NICU, where the baby often stays for a long time, may constitute a stressful experience, accompanied with fear and sense of impotence (Lundqvist and Jakobsson, 2003; Alderson et al., 2006; Lee et al., 2006; Lindberg et al., 2007), till manifestations of clinical importance, like anxiety and depression, that can have a negative impact on their relationship with the child (Feldman and Eidelman, 2007; Korja et al., 2008; Zelkowitz et al., 2011). In fact the premature birth and the consequent hospitalization of the newborn are moments of great importance with regard to the risk of postpartum depression in both parents: the separation from the infant, the fear for his survival, and their impotence in interacting with and taking active care of their child increase the risk of depressive symptoms (Davis et al., 2003).

Many studies pointed out that, compared with parents of full term infants, both mothers and fathers of premature infants showed significantly higher levels of depression and anxiety (Schmücker et al., 2005; Carter et al., 2007; Brandon et al., 2011). More recent research confirmed that paternal depression may affect the relationship between the mother and her baby (Ramchandani et al., 2005) and influence the emotional development and behavior of the child (Huhtala et al., 2011; Luoma et al., 2013); at the same time anxiety might compromise the paternal functions, the attachment process and the psychomotor development of the child (Luca and Bydlowski, 2001).

As the need to explore, also from a qualitative point of view, the impact of the preterm birth on both parents is increasingly clear, it is important the use of appropriate tools (such as interviews) to investigate parental perceptions, feelings, thoughts, and worries. However, on the basis of the existing literature, most research used only self-report questionnaires that are not sufficient to investigate deeply the “qualitative aspects” of the premature birth experience.

In a recent qualitative study (Hugill et al., 2013), where some semi-structured interviews were carried out with a group of fathers during the hospitalization of their premature infants, the results have shown that in most fathers their first meeting with the baby has provoked a feeling of a serious loss of emotional control. Their narratives have highlighted, moreover, the intense interior distress they encountered. They had often tried to react by hiding their emotions behind stereotypical behaviors in order to protect themselves from further pain. All the burden of the “silent emotional work” (Hugill et al., 2013) that they do during the hospitalization of their child should not be belittled.

Few studies, however, are based on interviews and, among them, an useful tool that explores specifically parents’ experience after a preterm childbirth is the CLIP, developed by Meyer et al. (1993). The authors underline the clinical utility of the CLIP: the interview allows parents to reflect on their feelings and concerns and it may be helpful for clinicians to determine “how psychosocial support services might best be provided” (Meyer et al., 1993, p. 198). Using this instrument Keren et al. (2003) analyzed the relation between representations of mothers of preterm infants, hospitalized in NICU, and the quality of mother–infant interaction. This study showed that the CLIP was a useful clinical tool and a predictor of early disruptions in the mother–infant relationship at the nursery. More recently two studies referred to this instrument. In the first, Tooten et al. (2013) used the CLIP to compare experiences and perceptions in parents of term, moderately and very preterm infants. In line with literature, this study highlighted that parents of term infants had more positive experiences and perceptions of their newborns than parents of preterm infants; besides no significant differences were found between the emotional experience of mothers and that of fathers. In the second, Rossman et al. (2015) through an adapted version of the CLIP explored the development of maternal identity in first-time NICU mothers. The growing interest toward this interview, that seems to be confirmed by its increasing use, led us to realize a research aimed at adapting the CLIP to the Italian context and exploring its possible connections with aspects (as depression and anxiety), very relevant in parents of premature infants.

## Materials and Methods

### Aims

Our main aim was to explore fathers’ psychological reactions to preterm birth, as compared to mothers’ ones. Thus, we have considered both members of the parental couple, focusing on their reactions to their child’s hospitalization in NICU.

The first objective was to evaluate levels of anxiety and depression in both parents, also detecting the possible presence of significant differences between them.

Moreover, we aimed to test if the factorial model of the CLIP, found by Keren et al. (2003) on a sample of mothers, could be applied to Italian people and also if it could be applied to fathers. A subsequent purpose was to explore the presence of correlations among the CLIP and levels of anxiety and depression in fathers during hospital stay.

### Participants

All the consecutive parents whose child was hospitalized immediately after preterm delivery at the NICU of the Chieti University Hospital (Italy) between July and September 2014 were invited to take part to the study. Of the 40 parental couples invited, all women and 32 men (80%) agreed to participate. Less fathers than mothers accepted to take part to the study since males were spending a very few time in the NICU because of their job commitments. In the present paper, we have only considered those parents who decided to participate in the research as couples. All the children were born before the 37th week of gestation, were unaffected by genetic illnesses, neonatal deformities, or neurological damages clinically identifiable at birth.

### Procedure

The study was projected and realized according to all the ethical guidelines of the Italian Association of Psychology (AIP) and was approved by the Ethics Committee of the Department of Psychological Sciences of Chieti University. Access to the research site was permitted by the Director of the NICU of the Chieti University Hospital.

After being informed of the aims of the research project and after having signed the informed consent, every parent individually filled in a socio-demographic questionnaire, followed by two self-report instruments, i.e., the EPDS (Cox et al., 1987) in order to evaluate depressive symptoms, and the two forms of the STAI (Spielberger et al., 1970) to investigate state and trait anxiety symptoms. Afterward the Italian version of CLIP (Meyer et al., 1993) was carried out with mothers and fathers in an individual way. All the tools were administered by a trained clinical psychologist between 10 and 20 days after childbirth. We chose this period for administration of both questionnaires and interview on the basis of the consideration that several days had passed from delivery and both mothers (also those who had a urgent cesarean delivery) and fathers have had the opportunity to approach the newborns and develop the first interactions with them.

### Measures

The *socio-demographic questionnaire* was developed by the Authors of the study in order to assess the composition of the nuclear family, the educational background, the story of the couple’s relationship and general information about the course of pregnancy and causes of premature delivery.

The *EPDS* (Cox et al., 1987) is a self-report questionnaire composed of 10 items scored on a 4-point Likert scale (ranging from 0 “never” to 3 “always”) designed to assess postpartum depression. The total score can range from 0 to 30. In the present study we referred to a cut-off of 8/9 for mothers, as proposed by Benvenuti et al. (1999) and a cut-off of 5/6 for fathers, following Matthey et al.’s (2001) suggestion. Previous studies found that EPDS showed satisfactory psychometric properties (Eberhard-Gran et al., 2001).

The *STAI* (Spielberger et al., 1970) is composed of two 20-items self-report scales applied to measure two types of anxiety, a temporary condition of ‘State Anxiety’ (STAI-Y1, anxiety in a specific situation) and a more general and long-standing quality of ‘Trait Anxiety’ (STAI-Y2, anxiety as a general trait). Every item can be evaluated on a frequency scale (from 1 “almost never” to 4 “almost always”), yielding to a total score ranging from 20 to 80 for both forms. The STAI is widely used to asses anxiety symptoms not only in clinical but also in non-clinical samples and has considerable validity, internal consistency (Cronbach’s α = 0.86) and reliability (Spielberger et al., 1970). In this study we have used the Italian version validated by Pedrabissi and Santinello (1989), i.e., for the STAI-Y1 the three levels of the state anxiety were assigned according to the following cut-off scores: low level (scores lower than 31 for women and 29 for men); intermediate level (scores comprised between 31 and 46 for women and between 29 and 41 for men); high level (scores above 46 for women and 41 for men). For the STAI-Y2, the three levels were assigned as follows: low (scores lower than 34 for women and 29 for men); intermediate (scores between 34 and 49 for women and between 29 and 43 for men); high (scores higher than 49 for women and 43 for men).

The *CLIP* (Meyer et al., 1993) was originally developed in order to explore how parents of a preterm child feel and perceive their current situation. Its semi-structured nature offers the clinician a twofold advantage: on the one hand, it allows to explore parental experience in a systematic way; on the other, its flexibility enables the clinician to tailor the questions to the conversational flow, deeply exploring the parent’s emotional experience.

The CLIP, that requires about one hour to complete, analyzes eight main areas: “Infant’s Current Condition”, “Pregnancy Course”, “Labor and Delivery”, “Relationship with the Baby and Feelings as a Parent”, “Reactions to NICU Environment and Staff”, “Relationship with the Family and Social Support”, “Discharge and Beyond” and a final wrapping up.

The CLIP is audio-recorded and transcribed verbatim; the original Authors (Meyer et al., 1993) suggested coding the interview through a content analysis; afterward, Keren et al. (2003) developed a coding system to analyze the narrative style and the affective tone of the interview. Through an exploratory factor analysis, they identified two factors that were termed Readiness for Parenthood and Parental Rejection. The former refers to such items as sense of parenting, trust in the NICU staff, emotional readiness for discharge; in addition it includes “a well-organized narrative that integrates both negative and positive experiences into a well-formed discourse” (Keren et al., 2003, p. 104); the latter includes items such as unplanned pregnancy, a negative first reaction to pregnancy, no feeling of mutual recognition and poor richness of content of interview.

On the basis of the original interview, we translated and adapted the CLIP to the Italian context through the method of the back-translation (Brislin, 1970). One bilingual translator blindly translated the CLIP from the English language into Italian, and another bilingual back-translated it to English. Differences in the original and the back-translated versions were discussed and resolved by joint agreement of both translators.

In this research project, we referred to the coding system developed by Keren et al. (2003).

### Statistical Analyses

All statistical analyses (carried out with the Statistical Software for Social Sciences, SPSS, version 19) were conducted on raw scores, because these data reflect the real individual responses. The use of other type of data, for example data estimate, could modify results, since they could determine several differences at the individual level because there are many types of estimates methods. Moreover for each data estimates error variances should be computed.

In order to verify differences among parents in all the examined variables, several ANOVAs and χ^2^ statistics were conducted. Checking for the factorial structure of the CLIP in an Italian sample, we carried out an exploratory factor analysis following the Keren et al.’s study (2003).

## Results

The final sample consisted of 64 subjects, i.e., 32 couples of parents of premature infants. Sociodemographic characteristics of parents and information on infants’ birthweight and APGAR score are presented in **Table 1**.

**Table 1:** Characteristics of fathers, mothers, and infants.

	Fathers (*N* = 32)			Mothers (*N* = 32)		
	Mean	*SD*	Range	Mean	*SD*	Range
**Age (years)**	35.42	3.89	26–42.5	33.81	4.68	24–45.5
**Education (%)**						
Middle school	25.0			15.6		
High school	59.4			46.9		
University	15.6			37.5		
**Occupation (%)**						
Worker	43.8			30.5		
Self-employed	31.3			16.1		
Housewives				21.9		
Other occupation	24.9			31.5		
**Infant birthweight (g)**	1566.85	522.89				
**Infant APGAR score 5 min after delivery**	7	1.02	5–9			

According to the birth weight, 15.6% of our sample was born with an Extremely Low Birth Weight (i.e., <1000 g) and 25% with a Very Low Birth Weight (i.e., <1500 g); according to the gestational age at delivery (mean = 31.75 weeks; *SD* = 2.49), 9.4% may be considered extremely preterm (i.e., lower than 28 weeks) and 28.1% very preterm (i.e., lower than 32 weeks).

In order to check for significant differences between parents with respect to depressive and anxiety symptoms we conducted an ANOVA. Mean and standard deviation for each parent and the *F*-values for differences between the two groups are reported in **Table 2**. As we can see mothers showed a higher level of depressive and anxiety symptoms than fathers. All differences were statistically significant.

**Table 2:** Differences (ANOVA) between mothers and fathers on depressive and anxiety levels.

		Fathers (*N* = 32)		Mothers (*N* = 32)		*F*_(1,62)_	Significance
		Mean	*SD*	Mean	*SD*		
**Depression**	*EPDS*	5.63	3.98	12.61	5.56	27.419	*p < 0.001*
**Anxiety**	*STAI-Y1*	33.16	10.02	41.75	11.40	10.260	*p < 0.01*
	*STAI-Y2*	32.00	6.45	38.68	10.72	9.041	*p < 0.01*

*EPDS, Edinburgh Postnatal Depression Scale; STAI, State Trait Anxiety Inventory*.

As far as depressive symptoms, we divided the sample into two groups according to EPDS cut-off. For women, we referred to the cut-off of 8/9 (Benvenuti et al., 1999); instead, for men we referred to a lower cut-off, i.e., 5/6 (Matthey et al., 2001). We found that 68.5% of mothers and 37.5% of fathers showed a level above the risk threshold. We conducted an ANOVA between mother and father samples using these cut-off. Results showed a significant difference between groups [*F*_(3,60)_ = 7.91; *p* < 0.001]. From *post hoc* analysis (Tukey method) mothers above the cut-off showed higher scores than either not at risk mothers (*p* = 0.025) or not at risk fathers (*p* = 0.000). No significant differences emerged between the two groups of fathers.

With regard to anxiety symptoms, we divided the sample according to the score thresholds suggested in the Italian manual (Pedrabissi and Santinello, 1989) of the two forms of the STAI.

**FIGURE 1:**
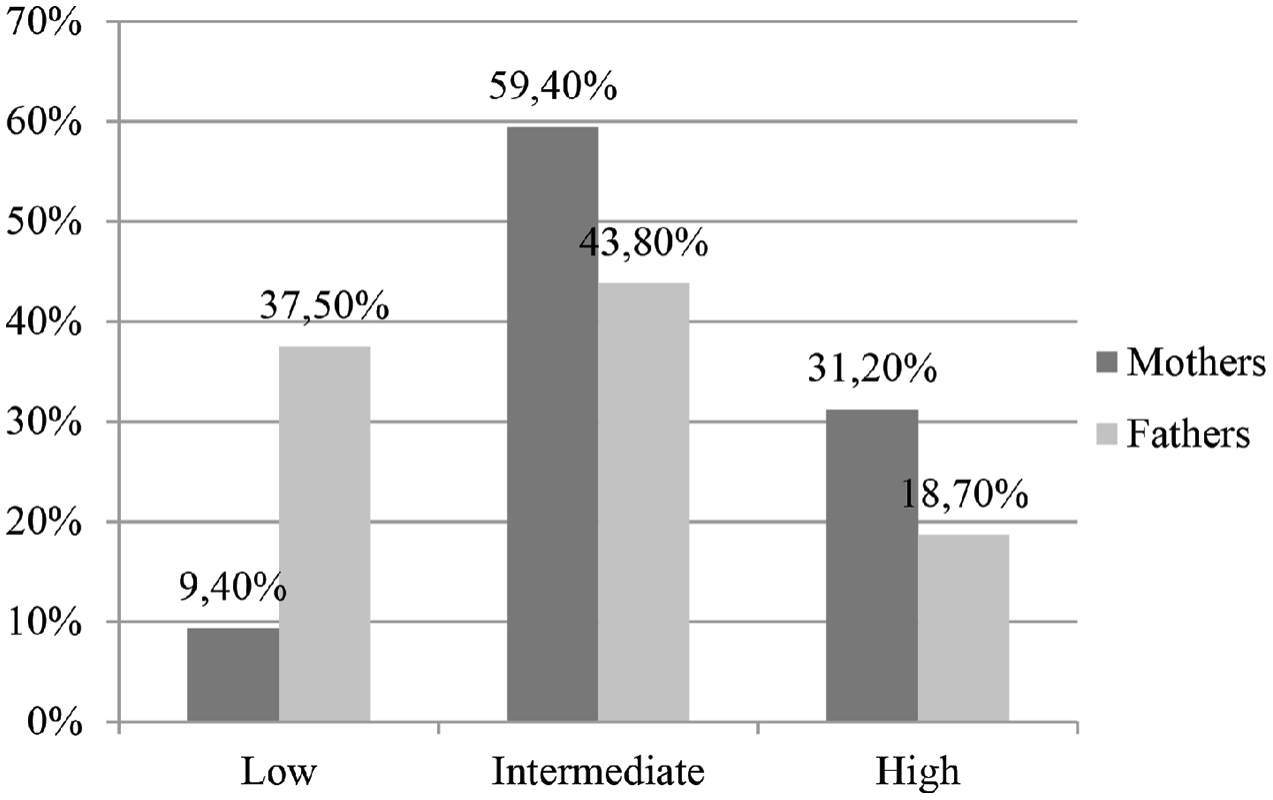
**Levels of state anxiety (STAI-Y1) in mothers and fathers**.

In **Figure 1** we synthesized mothers’ and fathers’ distributions for the State Anxiety. Most of the mothers and fathers showed an intermediate level, while a clinically significant state anxiety was found in 31.2% of the mothers and in 18.7% of the fathers. A χ^2^ statistic was used to assess the significant association between gender and level of state anxiety. The contribution of individual cells to association was examined through adjusted standardized residual. The overall χ^2^ statistic was significant [χ^2^_(2,63)_ = 14,269, *p* < 0.01]. Mothers and fathers were significantly different both in the low and intermediate categories of state anxiety but not in the high category.

**FIGURE 2:**
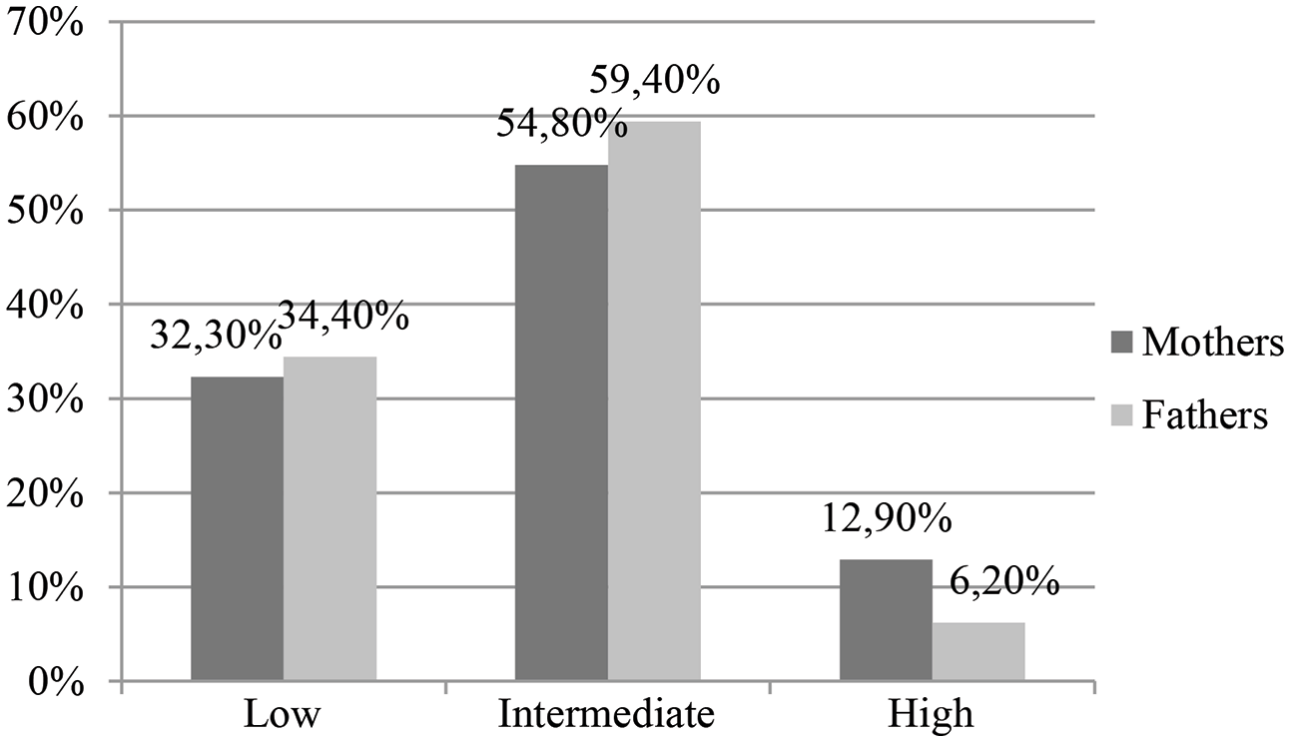
**Levels of trait anxiety (STAI-Y2) in mothers and fathers**.

Also for Trait Anxiety (**Figure 2**), the majority of both males and females were placed in an intermediate level while the percentage of subjects with a high level of symptoms was low. χ^2^ statistic was statistically significant [χ^2^_(2,63)_ = 8,461, *p* < 0.05]. Mothers and fathers differed in the low and high categories of trait anxiety, but not in the intermediate category.

As mentioned above, the CLIP is a relatively new tool in the Italian research context, so, first of all, we aimed to test its factorial structure. We carried out an exploratory factor analysis with principal component method and Varimax rotation. We would evaluate if the two factors (Readiness for Parenthood and Parental Rejection) found by Keren et al. (2003) may be applied to our sample of mothers and fathers.

With regard to the sample of mothers (see **Table 3**), the two factors explained together 38% of variance. This percentage is comparable with that found by Keren et al. (2003), i.e. 35%. Most of the items showed unique loadings on one factor, with the exception of three of them: e.g., Feeling of readiness for discharge, Discrepant expectations for baby’s future and Good support system. Most of the items loaded on Keren et al.’s factors. Namely on Readiness for Parenthood loaded Positive affect during interview, Fear of loss of premature baby, Confidence in NICU equipment, Positive present feelings toward baby, Feeling of readiness for discharge, Confidence in NICU staff, Confidence in self as parent of baby and Positive first feelings toward baby. Unexpectedly two more items loaded on this factor: negative first reaction to pregnancy and Unplanned pregnancy. On Parental Rejection loaded Poor richness of content of interview, Discrepant expectation for baby’s future and Lack of feeling of mutual recognition. Unexpectedly three items loaded on this factor: Readiness for birth, Good support system, and Well-organized narrative.

**Table 3:** Factorial structure of the maternal version of the CLIP.

	Factor 1	Factor 2
Positive affect during interview	**0.692**	
Fear of loss of premature baby	**0.619**	
Confidence in NICU equipment	**0.618**	
Positive present feelings toward baby	**0.614**	
Feeling of readiness for discharge	**0.607**	-0.337
Negative first reaction to pregnancy	**0.594**	
Confidence in NICU staff	**0.59**	
Confidence in self as parent of baby	**0.538**	
Unplanned pregnancy	**0.504**	
Positive first feelings toward baby	**0.469**	
Readiness for birth		**0.752**
Poor richness of content of interview		**0.694**
Discrepant expectations for baby’s future	0.33	**0.541**
Good support system	0.469	**0.493**
Lack of feeling of mutual recognition		**0.469**
Well-organized narrative		**0.401**
**% of variance**	**38.05%**	

*Bold values indicate each item mainly and greatest loadings on its specific factor*.

With respect to fathers, the exploratory factor analysis was less satisfactory, since the bifactorial structure explained only 28% of variance (**Table 4**).

**Table 4:** Factorial structure of the paternal version of the CLIP.

	Factor 1	Factor 2
Discrepant expectations for baby’s future	**0.762**	-0.371
Poor richness of content of interview	**0.584**	
Confidence in NICU equipment	**0.544**	
Fear of loss of premature baby	**-0.495**	
Confidence in NICU staff	**0.433**	
Lack of feeling of mutual recognition	**0.384**	
Confidence in self as parent of baby	**-0.335**	
Good support system		
Positive affect during interview	0.372	**-0.648**
Feeling of readiness for discharge		**0.639**
Positive present feelings toward baby	0.499	**0.531**
Negative first reaction to pregnancy	0.421	**0.458**
Well-organized narrative		**-0.433**
Unplanned pregnancy		**0.388**
Positive first feelings toward baby		
Readiness for birth		
**% of variance**	**28.54%**	

*Bold values indicate each item mainly and greatest loadings on its specific factor*.

As we can see, three items did not have significant loadings on the two factors: i.e., Good support system, Positive first feelings toward baby, and Readiness for birth. Moreover most of the items did not show a clear definition. As expected for Keren et al.’s Readiness for Parenthood factor, on Factor 1 loaded Confidence in NICU equipment, Fear of loss of premature baby, Confidence in NICU staff and Confidence in self as parent of baby, and unexpectedly loaded Discrepant expectations for baby’s future, Poor richness of content of interview and Lack of feeling of mutual recognition. On Factor 2 three of the six items showed a double loading: Positive affect during interview, Positive present feelings toward baby, and Negative first reaction to pregnancy. Then, as expected for Keren et al.’s Parental Rejection factor, only two items loaded on this factor: Negative first reaction to pregnancy and Unplanned pregnancy.

Given this unclear factorial structure, we decided to explore the presence of correlations among the CLIP items and levels of anxiety and depression in fathers. We carried out an analysis of correlations at the level of both two factors and single items of Keren et al.’s coding system. Results are showed in **Table 5**.

**Table 5:** Correlations of levels of depression and anxiety with CLIP items among fathers.

	EPDS	STAI_Y1	STAI_Y2
Fear of loss of premature baby	0.090	0.007	-0.074
Readiness for birth	-0.131	-0.189	-0.137
Positive first feelings toward baby	-0.019	0.222	-0.003
Positive present feelings toward baby	0.058	-0.030	0.041
Confidence in self as parent of baby	-0.079	0.114	0.051
Confidence in NICU staff	0.113	0.117	0.088
Confidence in NICU equipment	**0.375^∗^**	**0.408^∗^**	**0.427^∗^**
Good support system	-0.128	-0.198	-0.010
Feeling of readiness for discharge	-0.070	-0.162	0.081
Positive affect during interview	0.058	0.127	-0.142
Well-organized narrative	0.102	-0.148	-0.187
Negative first reaction to pregnancy	0.133	0.042	0.134
Unplanned pregnancy	**0.470^∗∗^**	0.107	0.318
Lack of feeling of mutual recognition	-0.053	-0.023	0.268
Discrepant expectations for baby’s future	0.261	-0.119	-0.029
Poor richness of content of interview	0.063	-0.151	0.044

*^∗^p < 0.05; ^∗∗^p < 0.01*.

As we can see, the only item that significantly correlated with both depressive and anxiety symptoms was Confidence in NICU equipment (*r*_EPDS_ = 0.375, *p* < 0.05; *r*_STAI-Y 1_ = 0.408, *p* < 0.05; *r*_STAI-Y 2_ = 0.427, *p* < 0.05). This item includes two prevalent aspects: on the one hand, the parents’ reaction to the NICU setting and, on the other, the reaction to their lack of control over baby. Higher levels of this item characterize parents with negative perceptions about the NICU equipment. Another CLIP item showed salient correlation only with depressive symptoms: Unplanned pregnancy (*r* = 0.470; *p* < 0.01).

## Discussion

Premature birth may represent a relevant risk factor that interferes in various ways with the normal duties and processes of parenthood.

Much has been written on the consequences that prematurity can produce on the psycho-physical growth of the child and on the building of the early parent–child bonds, but the majority of studies about preterm birth examined only the maternal point of view.

Overall our study aimed to explore parental emotional and affective reactions to a premature birth of a child, with a particular attention to fathers, since previous research often neglected the paternal perspective.

Our findings in general highlighted that fathers of premature child show a psychological distress that emerged through both self-report measures and clinical interview.

More specifically, with regard to our first purpose, fathers’ scores of anxiety were lower than mothers’ ones. However, if we refer to the state anxiety that could be the most influenced by the preterm birth situation, we would highlight that almost one fifth of males belonged to the high level category. Since recent studies (e.g., Ghorbani et al., 2014) comparing fathers of preterm and full-term infants found higher levels of anxious symptoms in the former, it could be important to pay attention to the paternal psychological experience immediately after a preterm childbirth.

In reference to depressive symptoms, scores observed in our sample of fathers were comparable to those relieved in a recent research (Helle et al., 2015) where the same questionnaire (EPDS) was used on a sample of parents of very low birth-weight infants one month after delivery. Our sample comprised not only very-low birthweight newborns but also low and extremely low birthweight ones, suggesting that preterm birth and the subsequent hospitalization in the NICU may produce an emotional impact on fathers regardless of the degree of risk. However, further research is needed in order to deepen this finding.

If we move to consider the EPDS cut-off among parents of both genders, we found that more than two thirds of the mothers and one third of the fathers exceeded the corresponding risk thresholds. For mothers this high incidence of depressive symptomatology after a premature birth is in line with the existing literature (Feldman and Eidelman, 2007; Brandon et al., 2011); conversely for fathers our data are worth of particular attention, especially in the light of the recent analysis on paternal depression (Paulson and Bazemore, 2010), which indicates that among the so-called ‘normal population,’ its prevalence immediately after the birth of a child is around 10.4%. If we compare this incidence with the percentage of fathers with depressive risk in our sample, we found that the latter is much higher and this may suggest a greater level of suffering among males after a premature delivery. These data were in line with previous studies (Carter et al., 2007; Mehler et al., 2014; Helle et al., 2015) that found significantly higher levels of depressive symptoms among fathers of premature babies than in fathers of full-term infants.

In reference to the difference between genders we may hypothesize that this finding can be connected to the fact that the woman, in relation to her partner, spends more time together with the child, especially during the hospitalization and feels a greater responsibility with regard to her role and her perception of not being directly involved in the care of her child (Östberg, 1998; Jackson et al., 2007).

In general the suffering caused by the premature birth of a child sets in motion intrapsychic processes and defense mechanisms in both parents which can produce a negative impact on the psychological wellbeing of the parental couple and on the child’s development. Recent studies confirmed that the presence of anxiety or depression in fathers may affect the paternal functions producing several consequences not only on the father–child relationship, but also on the development of the child and on the relationship between the mother and her baby (Ramchandani et al., 2005; Luoma et al., 2013).

Another purpose of this research was to analyze the factorial structure of the CLIP in our sample of mothers and fathers. With regard to mothers, the bi-factorial structure proposed by Keren et al. (2003) may be considered adequate, i.e., the Readiness for Parenthood and the Parental Rejection explained a percentage of variance comparable to the one observed in the original Keren et al.’s (2003) research.

Instead for fathers this structure seems to be less adequate. In fact many items showed double loadings and three items did not show loadings on neither of the two factors. The next step would be to find a new and greater sample of fathers in order to conduct an explorative and a confirmative factor analysis to identify the best fitting solution.

Another important aspect emerged from the correlation analysis between the CLIP and depression and anxiety measurements. In fact there was a significant correlation between the area of the CLIP called Confidence in the NICU equipment and the total scores of EPDS and STAI. The father seems to live the hospital situation with worries and feels depressed and anxious. It has been seen that the hospitalization of the newborn could have a very negative effect on the paternal emotional state: as Arockiasamy et al. (2008) found, the greatest causes of stress in fathers of preterm children admitted to an intensive care ward, are the environment itself of the NICU, the communication with the staff, the appearance of the child and the lack of preparation for a parental role which is completely different from the one that they imagined and desired during pregnancy. This same lack of preparation for the parental role could be addressed in order to explain the correlation we found between depression and unplanned pregnancy, in line with other studies (e.g., Leathers and Kelley, 2000). Further research could more deeply explore if an unplanned pregnancy may be considered a risk factor for the onset of depressive symptoms in fathers of premature infants.

Anyhow, the few significant correlations detected may depend on the use of methodologically different tools (i.e., a clinical interview and self-report instruments), thus in the future it could be interesting to compare the CLIP with other clinical interviews testing anxiety and depression.

All data collected in our research indicated the importance for nurses and clinicians working in the NICU to consider not only the maternal difficulties but also the paternal ones, even if these are often more hidden and silent (Hugill et al., 2013).

Our research, nevertheless, has some limitations. First, the small sample size may limit the generalizability of our findings. Second, self-report measures such EPDS and STAI may only indicate the level of anxious and depressive symptomatology without yielding to a clinical diagnosis. Third, the quality of this research (i.e., the integration of different methods such as questionnaires and interviews) makes it more difficult to interpret its results. However, this last limit may be also viewed as a strength: in fact, the integration of several tools made it possible to build up a more complete picture of the complex and difficult situation that parents (especially the fathers) of premature infants unexpectedly have to face.

Overall this study highlights the importance of paying attention to the experience of fathers in NICU. Becoming a father of a preterm born involves to play a stressful role, often characterized by depression and anxiety, as our results show. We would like to underline that our research opened up the possibility to carry on also other interesting reflections.

Through the clinical interview (CLIP), it has been possible to more deeply understand the emotional aspects linked to the experience of a premature birth for fathers, even if at times their narratives appeared to be “detached” or connected only to concrete features.

Contents of the CLIP have highlighted many themes among fathers (for example, those of anguish, fear of loss, feeling of distance, sense of alienation, feeling of unreality), in line with other research in NICU (Lundqvist et al., 2007; Arockiasamy et al., 2008). Many fathers mentioned that they sought to protect themselves from events that were too painful, hiding their real emotions; others affirmed that they felt themselves drawn to hide their emotions in order to protect their partner. Besides, during the administration of the CLIP, clinical trained psychologists who carried out the interview, because of their psychotherapeutic background, were able to notice some defense mechanisms as denial and rationalization used to deal with the distress situation they were experiencing. These defense mechanisms were seen also in fathers with anxiety and depressive levels under the risk thresholds; this observation let us to highlight the importance to combine the administration of self-report instruments with the use of clinical interviews carried out by trained psychologists, in order to more deeply understand the suffering of fathers.

We think that fathers and mothers of premature infants equally need to be helped immediately after the childbirth and the subsequent hospitalization, since early interventions focused on parents may be able to prevent future difficulties in their relationship with infant and in child’s development (Herd et al., 2014; Mortensen and Mastergeorge, 2014).

The CLIP may be considered an useful interview not only for research purposes but also for clinical ones for stimulating parents to reflect on their stressful experience in an empathic context and our study underlines the utility to deepen its use among fathers.

## Conflict of Interest Statement

The authors declare that the research was conducted in the absence of any commercial or financial relationships that could be construed as a potential conflict of interest.

## Abbreviations

ANOVA: analysis of variance
CFA: confirmatory factor analysis
CLIP: Clinical Interview for Parents of High-Risk Infant
EPDS: Edinburgh Postnatal Depression Scale
NICU: Neonatal Intensive Care Unit
STAI: State Trait Anxiety Inventory
